# Fabrication of Silk Fibroin/Graphene Film with High Electrical Conductivity and Humidity Sensitivity

**DOI:** 10.3390/polym11111774

**Published:** 2019-10-28

**Authors:** Haoran Zhang, Juntao Zhao, Tieling Xing, Shenzhou Lu, Guoqiang Chen

**Affiliations:** National Engineering Laboratory for Modern Silk, Soochow University, Suzhou 215123, China; 20174215030@stu.suda.edu.cn (H.Z.); 20185215009@stu.suda.edu.cn (J.Z.); chenguojiang@suda.edu.cn (G.C.)

**Keywords:** silk fibroin, graphene, flexibility, electrically conductive, humidity sensitive

## Abstract

Silk fibroin (SF) is a natural material with good biocompatibility and excellent mechanical properties, which are complementary to graphene with ultrahigh electrical conductivity. In this study, to maximally combine graphene and silk fibroin, a well-dispersed silk fibroin/graphene suspension was successfully prepared in a simple and effective way. Then we prepared a flexible conductive SF/graphene film with a minimum resistance of 72.1 ± 4.7 Ω/sq by the casting method. It was found that the electrical conductivity of the SF/graphene film was related to the water content of the film, and the variation was more than 200 times. Therefore, it will play an important role in the field of humidity sensors. It also has excellent mechanical properties in both wet and dry states. These unique features make this material a promising future in the fields of biomedical applications, wearable sensors, and implantable internal sensors.

## 1. Introduction

Flexible and wearable electronics have great potential to be applied in human health monitoring systems [1]. They were made into a variety of sensors to monitor human health, such as strain, pressure, temperature, humidity, and electrochemical sensors [2,3,4,5,6]. Even though they have their multifunctional and powerful monitoring capabilities, they still cause skin discomfort or even rejection during prolonged wear. Therefore, it is necessary to improve the skin adaptability, comfort, and biocompatibility. Silk fibroin, as a natural macromolecule material, is a good choice for improving skin adaptability and biocompatibility. Due to good biocompatibility, degradability, and excellent mechanical properties, it has been widely used in medical fields, such as surgical sutures, biological scaffolds, artificial blood vessels, and microneedles [7,8,9,10,11]. However, the silk fibroin is insulating and need to combine with some conductive materials such as ionic liquid [12,13], conducting polymers [14,15], and carbon materials [16,17], to increase the electrical conductivity of the silk materials for the application in the biosensors field [18,19]. As one of the carbon materials, graphene has become one of the most important materials since it was successfully separated from graphite by using a “scotch-tape” method in 2004. Graphene has thermal, electrical, and mechanical properties [20,21,22] due to its 2D sp^2^ carbon honeycomb structure. Meanwhile, SF/graphene composite materials also have good biocompatibility. Vera-Sanchez et al. [23] cultured periodontal ligament stem cells on SF/ graphene oxide (GO) and SF/reduced graphene oxide (RGO) membranes, and the results showed that SF/RGO membrane was more favorable for the differentiation of periodontal ligament stem cells into cementoblasts. Wang et al. [24] prepared a biocompatible SF/RGO composite film by solution casting to promote cell adhesion, growth, and proliferation.

Accordingly, we intend to combine graphene and silk fibroin to prepare flexible and wearable electronics with good skin comfort. However, it is challenging to disperse graphene in silk fibroin [25,26] and to keep the balance between the mechanical and electrical properties. Previous studies reported that the preparation of SF/graphene composite materials could be divided into the following: the graphene feeding method, the SF/graphene oxide reduction method, and the SF/graphene solution method. Firstly, the graphene feeding method is the pioneering method to successfully combine silk fibroin and graphene materials, which provides a solid foundation for the development of subsequent silk fibroin/graphene composite materials. Wang et al. [27] reported mechanically enhanced silk directly collected by feeding *Bombyx mori* larval silkworms with graphene. Secondly, it is well known that graphene is highly hydrophobic and chemical inert, and cannot be dispersed in silk fibroin solution, but graphene oxide (GO) can be dispersed. Therefore, GO is mixed with the silk fibroin (SF) and then reduced to obtain the graphene/SF composite material. Wang et al. [28] prepared graphene and silk fibroin-based carbon (GCN-S) materials by carbonization of reduced graphene oxide (RGO) and silk fibroin nanofibrillar composites in the presence of KOH and the materials have excellent electrochemical performance. However, in addition to high-temperature carbonization, it is difficult to completely reduce GO to RGO by general a reduction method, such as chemical reduction. Excessive temperature is too harsh for most materials. Moreover, due to defects in the redox process, the conductivity of RGO is much lower than that of pristine graphene. Thirdly, another preparation way is to improve the dispersibility of graphene in water. Wang et al. [24] just dispersed RGO in a silk fibroin solution by a weak ultrasonication and cast film on the Polydimethylsiloxane (PDMS)substrate. This simple dispersion method is based on the fact that there are still a certain number of hydrophilic groups in RGO, and the dispersibility in water is better than that obtained by the stripping method. Furthermore, the RGO has weak electrical conductivity, which may be the reason the electrically conductive properties of composite materials do not mention in this work. Ling et al. [18] used a kind of 3D printing graphene ink [29] to prepare Graphene/SF/HFIP and Graphene/SF/FA/Ca^2+^ suspensions, which are suitable for the fabricateion of polymorphic materials, such as fibers, films, and coatings. On Ling’s basis, Wang et al. [30] made an electronic tattoo that is responsive to environmental changes, such as strain, humidity, and temperature variations. It’s really amazing work, but a lot of toxic substances were introduced into the process, and it should be purer. Even so, the third method is obviously better. 

Therefore, a safe, mild, and non-toxic dispersant is urgently needed, and poly (vinyl alcohol) (PVA) is a good choice [31,32,33]. PVA has an amphiphilic structure which can disperse the graphene aqueous solution. Meanwhile, PVA also has such good biocompatibility that is widely applied in the field of medical supplies [34,35]. Butanediol is a common moisturizer in the cosmetics field and has good biocompatibility. The addition of butanediol to the silk fibroin can induce silk fibroin to form a crystalline structure, which can significantly reduce the dissolution rate of the silk fibroin film and increase the flexibility of the film [36].

In this work, butanediol was used to improve the flexibility of the silk fibroin, and an appropriate concentration of graphene/PVA suspension was prepared. Then, the two suspensions were mixed, and the silk fibroin/graphene composite film was obtained by the casting method. This film material made by the method we proposed has excellent electrical, and mechanical properties and it is expected to be applied in flexible and wearable electronics, humidity sensing, and biomedical field. 

## 2. Materials and Methods

### 2.1. Materials

Silkworm (*Bombyx mori*) cocoons were purchased from Suzhou Siruibao Biotechnology Co., Ltd. (Suzhou, China). Graphene/*N*-methyl-2-pyrrolidone (NMP) (1–8 layers, 0.2–3 μm) solution was purchased from Shanghai Najiu Intelligent Technology Co., Ltd. (Shanghai, China). Cellulose dialysis membrane (8000–14,000 Da) was bought from Union Carbide Corporation (Houston, TX, USA). PVA-103 was provided by Aladdin Industrial Corporation (Shanghai, China). Sodium carbonate was purchased from Sinopharm Chemical Reagent Co., Ltd. (Beijing, China). 1,4-Butanediol was supplied by Shanghai Lingfeng Chemical Reagent Co., Ltd. (Shanghai, China). Lithium bromide was purchased from Tiancheng Chemical Co., Ltd. (Jining, China). All the chemicals were of analytical reagent grade and used without further purification. DC power supply (MT-1520) was bought from Maisheng Power Technology Co., Ltd. (Guangzhou, China).

### 2.2. Preparation of SF/Graphene Film

#### 2.1.1. Preparation of the SF Solution

The silkworm cocoons were degummed in a 0.02 mol/L Na_2_CO_3_ boiling water solution for 30 min, three times, and dried in an oven at 60 °C. The degummed SF was dissolved in 9.3 mol/L LiBr solution at 65 °C for 1 h followed by dialysis against deionized water for three days using cellulose dialysis membrane (8000–14000 Da) with frequent water changes.

#### 2.1.2. Preparation of the SF/Butanediol Solution

A 10% (*w*/*v*) solution of butanediol (BD) was added to the silk fibroin solution at a mass ratio of SF: Butanediol = 5:1 to obtain a SF/BD mixed solution.

#### 2.1.3. Preparation of Graphene/PVA(GrP) Suspension

PVA was dissolved in deionized water at 95 °C for 3 h, and then the solution was cooled to room temperature. The NMP solution of graphene was diluted with a certain amount of deionized water, and a certain amount of 10% (*w*/*v*) PVA aqueous solution was added according to the mass ratio Graphene: PVA = 4:1. The dispersion was then dialyzed with deionized water for 48 h to obtain Graphene/PVA dispersion. The concentration of the graphene dispersion is 2 mg/mL.

#### 2.1.4. Preparation of the SF/Graphene Film

**Figure 1:**
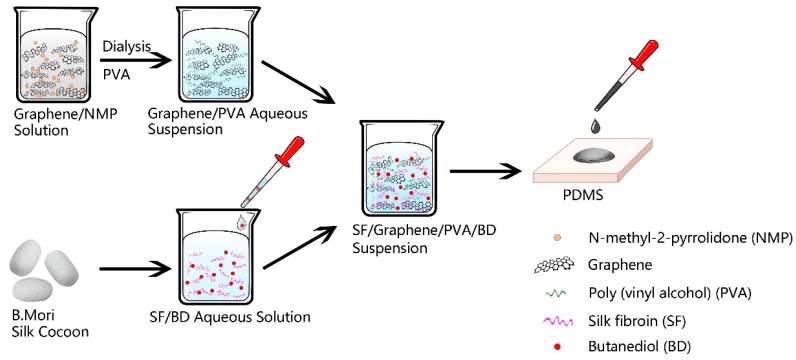
Flow chart for preparation of SF/Graphene film.

The prepared SF (or SF/BD) solution and the Graphene/PVA dispersion were uniformly mixed according to the mass ratio, and the Graphene/SF/PVA mixed solution was dropped on the PDMS template, and air-dried at 20 °C and 65% relative humidity (RH). Thus the SF/Graphene film was obtained. (Figure 1)

### 2.3. Characterization of the SF/Graphene Film

#### 2.3.1. Cross-sectional Morphology

The Hitachi S-4800 scanning electron microscope (SEM) was used to observe the cross-sectional morphology of SF/GrP/BD film (SF:GrP:BD = 10:2:1) at an acceleration voltage of 3 kV.

#### 2.3.2. XRD

For sample preparation, SF, SF/BD, SF/GrP, SF/GrP/BD films (SF:BD = 5:1, SF:GrP = 10:1, SF:GrP:BD = 10:2:1) were cut into powders. The crystal image of the sample was analyzed by the X’pert-Pro MRD X-ray diffractometer (XRD) (Panaco, the Netherlands). Test condition: tube current 35 mA, tube voltage 40 kV, scanning speed 5 °/min and using Cu–Kα rays. XRD patterns were recorded in the 2θ region from 5° to 40°.

#### 2.3.3. ATR-FTIR

Attenuated total reflectance Fourier transformed infrared spectroscopy (ATR-FTIR) was used to analyze the structural changes of the SF in four kinds of films (the same as XRD). All infrared spectra were recorded in the range of 4000–550 cm^−1^ using the Nicolet iS5 spectrometer, equipped with an iD7 ATR accessory (Thermo Scientific, Waltham, MA, USA). Each spectrum was acquired by the accumulation of 32 scans with a resolution of 4 cm^−1^.

#### 2.3.4. Dissolution Rate

Different SF/GrP/BD film samples were taken and placed in an oven at 90 °C for 3 h to test *M* as the weight after drying. The film was then placed in 20 mL of deionized water and shaken in a 37 °C water bath for 1 h. The absorbance was measured for the solution using an ultraviolet-visible spectrophotometer at 278 nm to evaluate the concentration (*C*) of silk fibroin dissolution in deionized water, and the film loss rate was calculated according to the following formula [37]:

(1) $$D(\%)=\frac{M-CV}{KM}\times 100\%$$

where, *D* is the dissolution rate, *M* is the weight after drying, *C* is the silk fibroin concentration obtained from the absorbance comparison standard curve, K is the percentage of silk fibroin content in dry weight of the film, and V is the total volume of the solution.

#### 2.3.5. The Effect of the Water Content of Films on Sheet Resistance

The water content of the SF/GrP/BD film was tested as follows. The film was firstly immersed in deionized water for 24 hours. After taking out, the water droplet on the film surface was absorbed with absorbent paper. The saturated water content and mass swelling rate (water) were calculated and the sheet resistance was measured. Then, during the film drying process, the water content, and the sheet resistance were tested at different times. When the quality of the film remained unchanged, it was placed in an oven at 90 °C and dried for 3 h, which was recorded as moisture content of 0%. In this process, the water content and the sheet resistance were tested separately at various times. Repeat the above process three times for the same film. At a certain moment, weigh the film mass to *M*1, and this film was placed in an oven at 90 °C for 3 h to be taken out and weighed, *M*2. Then the real-time water content of the film at that moment is E. The film’s mass swelling rate (water) is S.

(2) $$E(\%)=\frac{M1-M2}{M1}\times 100\%$$

(3) $$S(\%)=\frac{M1-M2}{M2}\times 100\%$$

The conductivity of the films was compared by measuring the sheet resistance of the film. The sheet resistance of the sample was tested with the ST-2258C multi-function digital four-probe tester, and five points for each film were tested to calculate the average value and variance.

#### 2.3.6. Mechanical Properties

The mechanical properties of the films were tested by a BOSE ELF3220 machine (BOSE, USA) in tensile mode at 20 °C and 65% relative humidity with a tensile speed of 0.008 mm/s. The SF, SF/BD, SF/GrP, and SF/GrP/BD films were cut with a dimension of 5 mm × 10 mm × 50 μm and placed in a constant temperature and humidity (20 °C and 65% (RH)) room for 24 hours for dry mechanical testing. For wet testing, films were soaked in deionized water for 24 h at room temperature. Subsequently, samples for wet testing were loaded into the tester and the surface dried with absorbent paper. The clamping distance and tensile speed were 8 mm and 20 mm/min, respectively. Each sample was repeated six times.

#### 2.3.7. Water Rate LED Lamp Bead Test and Flexible dDsplay

A simple series circuit was built with 3 W Light-Emitting Diode (LED) lamp bead, the SF/GrP/BD film (cut with a dimension of 5 mm × 10 mm × 50 μm and placed at a constant temperature and humidity (20 °C and 65% relative humidity) for 24 hours), DC power supply, and wire. Once the power switch is turned on, and the small bulb starts to light, then 3 mL deionized water is dropped onto the SF/GrP/BD film with a dropper. The brightness change of the small bulb is then observed within the next three minutes [30].

## 3. Results and Discussion

### 3.1. Effect of Different Ratios of PVA on Graphene/PVA Suspension

Graphene cannot form a stable aqueous solution alone due to its strong hydrophobicity. Many reports mentioned that PVA could solve the problem of graphene water solubility [28]. On the one hand, PVA molecules can be well adsorbed on the surface of graphene sheets. On the other hand, PVA has a large number of hydroxyl groups to make it hydrophilic, so that PVA-coated graphene can be well dispersed in aqueous solution. 

**Figure 2:**
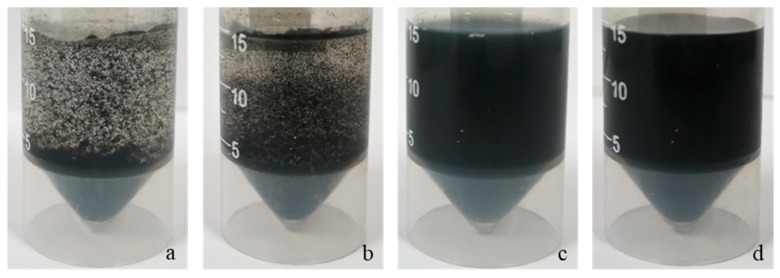
The images of Graphene/PVA suspension with different solid content ratios of (**a**) pure graphene, (**b**) 100:1, (**c**) 10:1, (**d**) 1:1 (*w*/*w*).

Figure 2 shows the images of Graphene/PVA suspension with different solid content ratios. When the ratio of pure graphene and Graphene/PVA is 100:1, a homogeneous dispersion cannot be formed in water, and obvious agglomerated graphene particles can be seen (Figure 2a,b). In contrast, Graphene/PVA = 10:1 and 1:1 are both homogeneous suspensions, and almost no graphene particles are visible (Figure 2c,d). It was confirmed that PVA has a good dispersion effect on graphene. Therefore, considering the huge difference between 100:1 and 10:1, and the inconspicuous difference between 10:1 and 1:1, we finally chose Graphene/PVA = 4:1 for the next work.

### 3.2. Cross-Sectional Morphology

**Figure 3:**
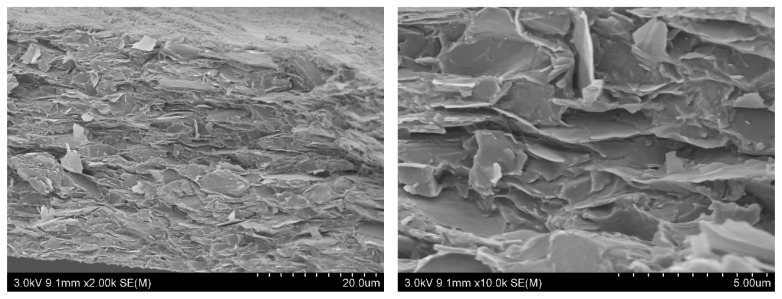
Cross-sectional morphology of SF/GrP/BD films (SF:GrP:BD = 10:2:1).

Figure 3 shows the cross-sectional morphology of SF/GrP/BD films. The layered structure with a relatively neat orientation can be obviously observed. Broken graphene sheets could also be seen in some places. These results confirm the good dispersion of graphene and silk fibroin in the film. This regular structure also ensures the electrical conductivity and mechanical properties of the film to some extent.

### 3.3. XRD and ATR-FTIR

**Figure 4:**
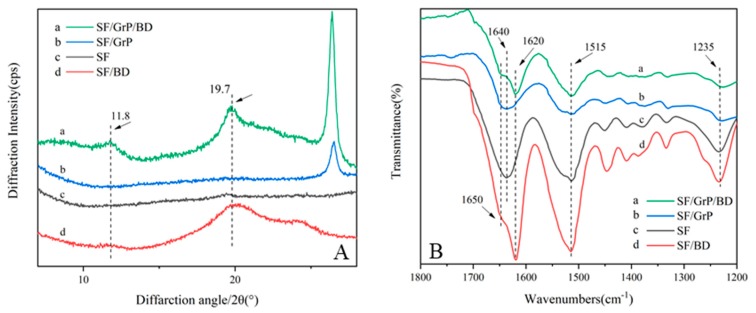
(**A**) XRD and (**B**) ATR-FTIR patterns of SF, SF/BD, SF/GrP, SF/GrP/BD films.

In order to investigate the structural changes of SF after loading GrP and BD into the film, XRD and ATR-FTIR tests were performed (Figure 4). From the XRD figure, it can be seen that the pure SF has no obvious peaks, indicating that pure SF mainly exists in the form of random-coil (Figure 4A, c). After adding BD alone, there is a strong peak at 19.7° and a weaker peak at 11.8°, indicating a mainly silk I crystal structure (Figure 4A, d) [36]. In contrast, the addition of GrP alone has no effect on the structure of SF, and only a characteristic peak belonging to graphene appears at 26.5° (Figure 4A, b). This indicates that graphene has no effect on the structure of SF, and the change of SF structure is predominantly derived from the influence of BD. 

SF has three most characteristic amide regions in the infrared spectrum, namely amide I (1700–1600 cm^−1^), amide II (1600–1500 cm^−1^), and amide III (1350–1200 cm^−1^). From the ATR-FTIR figure, it can be seen that SF and SF/GrP both have absorption peaks at 1640 cm^−1^, corresponding to the characteristic peaks of the random-coil (Figure 4B, b,c). The absorption peaks of SF/BD and SF/GrP/BD at 1650 cm^−1^ and 1620 cm^−1^ are characteristic peaks of α-helix and β-sheet, which are consistent with the result of XRD (Figure 4B, a,d) [36]. Meanwhile, it can be found from the ATR-FTIR spectrum, graphene has little effect on the spectrum, except that some transmittance decreases due to the color changes of the film (from colorless transparency to black). This is because graphene itself has no activity on FTIR and no effect on the structure of SF. In summary, the secondary structure change of SF is only related to BD in this system.

### 3.4. Dissolution Rate

**Figure 5:**
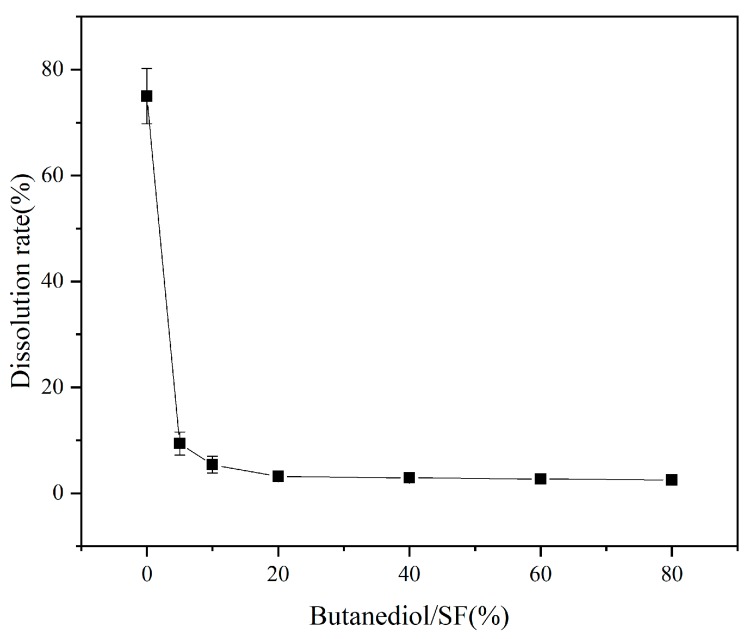
The dissolution rate of BD in different proportions of SF (*w*/*w*).

Figure 5 shows the change of protein dissolution rate of SF/GrP/BD films with BD content ranging from 0% to 80% in SF (*w*/*w*). Since SF without BD mainly exists in the film in the form of a random-coil, the dissolution rate is as high as 76.5%. When the BD/SF (%) is 5%, the dissolution rate drops to 9.7%. With the increase of BD content, the dissolution rate of the film decreases gradually. When the BD/SF (%) is 20%, the dissolution rate of the film decreases slightly, and it was basically stable at about 3%. Therefore, the addition of BD could solve the water solubility problem of SF/GrP film materials. 

### 3.5. Water Content and the Sheet Resistance

**Figure 6:**
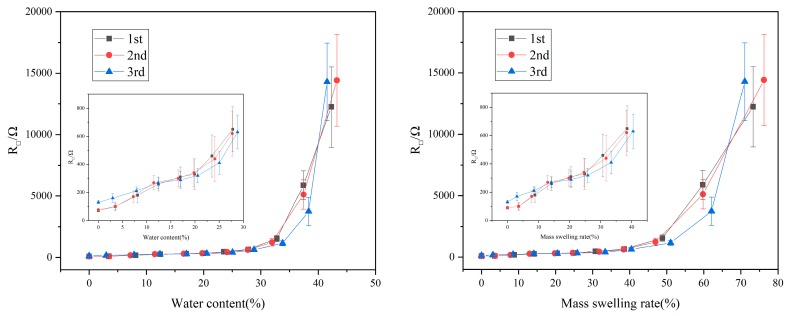
Relationship between water content/mass swelling rate and R_□_ of the SF/GrP/BD film repeatedly absorbing moisture three times.

**Figure 7:**
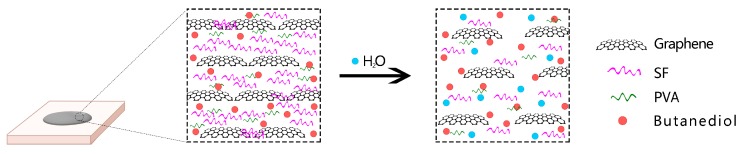
Swelling mechanism of SF/GrP/BD Film.

The properties of many polymer materials are strongly related to their water content [38]. Therefore, the effect of water content cannot be ignored in the study of the properties of such materials. As shown in Figure 6, the sheet resistance of the film increases with the increase of water content and mass swelling rate. The mass swelling ratio and the water content are obtained by different calculations using the same data; therefore, the subsequent relationship between the mass swelling ratio and the sheet resistance is not discussed separately. When the water content is 0%, the minimum resistance of the film is 72.1 ± 4.7 Ω/sq, and when the water content is close to 42% (the saturated water content), the sheet resistance is about 15 kΩ/sq. In other words, the resistance of film varies about 200 times from the saturated water content to 0% water content. At the same time, the film resistance changes steadily after three consecutive water absorption and drying processes. The change in electrical resistance is mainly due to the increase of water content, and the swelling of the SF results in the increase of the distance between the graphene sheets (Figure 7). When the water content is lowered, and the silk fibroin is restored, the distance between the graphene sheets is also restored, and the electric resistance is lowered. Therefore, we believe that this material has great potential in the field of moisture-sensitive materials [39,40].

### 3.6. Mechanical Properties

The dry and wet mechanical properties of SF, SF/BD, SF/GrP, SF/GrP/BD films are shown in Table 1 (due to the high dissolution rate of SF and SF/GrP films, the wet mechanical properties could not be obtained). By comparing SF and SF/BD films, it can be found that the addition of BD acts as a plasticizer, transforming the pure SF film from a brittle material to a ductile material. After the addition of GrP, the strain notably decreased from 6.59 ± 1.55% to 1.11 ± 0.21% and the modulus increased from 1.8 ± 0.2 GPa to 5.3±0.7 GPa. The results show the SF/GrP film has higher rigidity than SF films due to the ultrahigh stiffness of graphene itself and the synergistic effect of SF and graphene. Comparing the dry and wet properties of the same film, it was found that the tensile strength and modulus of the film decreased in the wet state, and the strain increased greatly. This indicates that with the increase of water content, water molecules also act as plasticizers, promoting the transformation of materials to ductile materials [30]. Finally, the SF/GrP/BD film has excellent mechanical properties in both wet and dry conditions with a good balance between stiffness and toughness. Customizable mechanical properties could be obtained by changing the content of GrP and BD in the later work.

### 3.7. Water Rate LED Lamp Bead Test and Flexible Display

**Figure 8:**
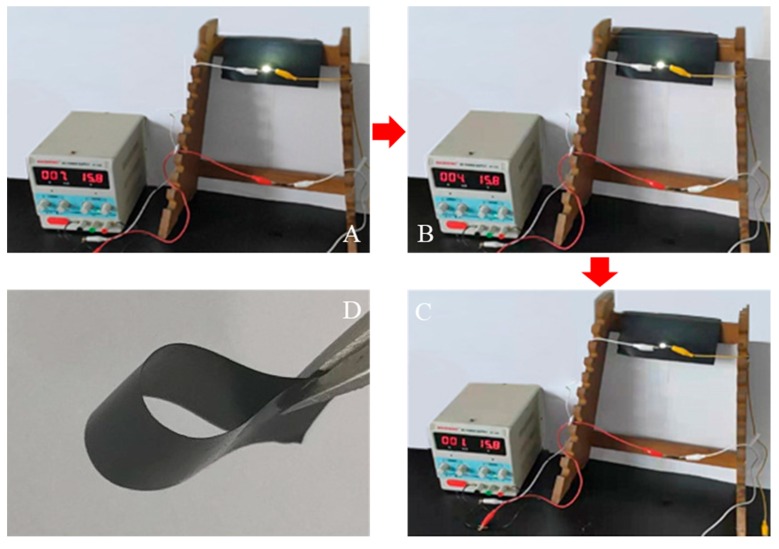
(**A**) 0 s, (**B**) 60 s, and (**C**) 170 s of the LED lamp bead brightness picture after dripping, respectively. (**D**) Flexible display of the SF/GrP/BD film.

According to the above result of the sheet resistance changes with water content, the lower the water content, the lower the sheet resistance. Therefore, when a droplet was dropped onto the film, the film resistance became large, and the LED lamp bead gradually darkened. It can be seen from Figure 8A–C that the LED lamp bead gradually darkens within 170 s after dripping and the current value displayed by the DC power supply (7 mA in 0 s, 4 mA in 60 s, and 1 mA in 170 s). The LED lamp bead brightness had obvious differences between A and C. A gradual decrease in the current verified the previous result. 

## 4. Conclusions

In this study, a flexible conductive SF/Graphene composite film with high conductivity (minimum resistance of 72.1 ± 4.7 Ω/sq) was successfully prepared by a simple combination of SF and graphene. The addition of BD solved the problem of water solubility of the film and improved the toughness. The structure of the film was characterized by SEM, XRD, and ATR-FTIR. The results showed the obvious and relatively neat orientation of the layer structure of graphene sheets, and the addition of BD changed the secondary structure of the silk fibroin, which reduced the dissolution rate of the film. The dissolution rate of the SF/GrP/BD films with different BD content was determined, which laid a foundation for the subsequent wet state experiment. It was found that the resistance of the film had a great relationship with the water content, which was caused by the increase of the distance between the graphene sheets derived from the swelling of SF. The change variation was more than 200 times, and the repeatability is good, far exceeding the ordinary humidity sensor. The SF/Graphene composite film had excellent mechanical properties in both wet and dry states, which can meet the needs of various situations. For demonstration purposes, we utilized SF/GrP/BD films for LED lamp bead test and flexible display. These attractive performances encourage further exploration of their applications in the fields of biomedical materials, wearable sensors, and implantable internal sensors. 

## Figures and Tables

**Table 1:** Dry and wet mechanical properties of SF, SF/BD, SF/GrP, and SF/GrP/BD films.

Sample.	Stress [MPa]	Strain [%]	Modulus [Gpa]
SF	55.1 ± 6.8	6.59 ± 1.55	1.8 ± 0.2
SF/BD	33.4 ± 3.2	68.21 ± 22.76	0.6 ± 0.1
SF/GrP	28.6 ± 2.4	1.11 ± 0.21	5.3 ± 0.7
SF/GrP/BD	12.3 ± 1.7	11.41 ± 2.62	0.9 ± 0.1
SF/BD (wet)	9.2 ± 1.9	182.37 ± 45.48	0.04 2± 0.009
SF/GrP/BD (wet)	5.6 ± 0.7	155.88 ± 38.27	0.063 ± 0.017
